# RVG29-modified microRNA-loaded nanoparticles improve ischemic brain injury by nasal delivery

**DOI:** 10.1080/10717544.2020.1760960

**Published:** 2020-05-13

**Authors:** Rubin Hao, Bixi Sun, Lihua Yang, Chun Ma, Shuling Li

**Affiliations:** a Institute of Traditional Chinese Medicine, Changchun University of Chinese Medicine, ChangChun, Jilin, China;; b School of Pharmaceutical Sciences, Jilin University, ChangChun, Jilin, China;; c Affiliated Hospital of Changchun University of Chinese medicine, ChangChun, Jilin, China

**Keywords:** Nose-to-brain, Ischemic brain injury, miRNA, RVG29

## Abstract

Effective nose-to-brain delivery needs to be developed to treat neurodegenerative diseases. Regulating miR-124 can effectively improve the symptoms of ischemic brain injury and provide a certain protective effect from brain damage after cerebral ischemia. We used rat models of middle cerebral artery occlusion (t-MCAO) with ischemic brain injury, and we delivered RVG29-NPs-miR124 intranasally to treat neurological damage after cerebral ischemia. Rhoa and neurological scores in rats treated by intranasal administration of RVG29-PEG-PLGA/miRNA-124 were significantly lower than those in PEG-PLGA/miRNA-124 nasal administration and RVG29-PLGA/miRNA-124 nasal administration group treated rats. These results indicate that the nose-to-brain delivery of PLGA/miRNA-124 conjugated with PEG and RVG29 alleviated the symptoms of cerebral ischemia-reperfusion injury. Thus, nasal delivery of RVG29-PEG-PLGA/miRNA-124 could be a new method for treating neurodegenerative diseases.

## 1. Introduction

The treatment of cerebral apoplexy has always been clinically difficult, and the effects of conventional oral and intravenous treatments are unsatisfactory. The focus of cerebral apoplexy treatment is to protect normal brain tissue and promote the recovery of the ischemic penumbra near the infarcted area (Leigh et al., 2018). After cerebral infarction, the morphological structure of nerve cells changes, leading to a loss of function, but functional recovery can occur after a short period (Muzzi et al., 2019). Currently, the commonly used treatment method in clinical practice is chemical drug thrombolysis, but the effect is insufficient. Nucleic acid drugs have good pharmacodynamic effects, low toxicity, and few side effects. At present, treatment with nucleic acids has received increasing attention (Davide et al., 2019). MicroRNAs (miR) are short single-stranded RNAs with specific regulatory functions that can control signal pathways through a related network (Adlakha & Saini, 2014). MiR-124 is one of the most abundantly expressed miRNAs in the mature central nervous system (CNS) and is closely related to neuronal differentiation, maturation, and survival (Kozuka et al., 2019). In preclinical studies of neurological diseases, miR-124 is often used to assess neuroprotection and functional recovery after cerebral apoplexy (Hamzei Taj et al., 2016), and this molecule plays an important role in the plasticity of synaptic homeostasis (Hou et al., 2015).

In terms of drug delivery for the treatment of cerebral ischemia, nanoparticles are formed by polymer-encapsulated chemical drugs, and the blood-brain barrier is bypassed by nasal administration, which has gradually gained public acceptance (Sonvico et al., 2018). An increasing number of preclinical studies have confirmed that a nasal inhalation system for drug delivery is a safe, noninvasive and convenient delivery strategy (Karthivashan et al., 2018) and can be used as an alternative to conventional administration routes that cannot cross the blood-brain barrier (Mittal et al., 2014). Nanoparticles formed by miR-124 can protect nucleic acids from degradation by proteases. Nucleic acids can enter the brain directly from the nose along the olfactory and trigeminal pathways by nanoparticles (Sukumar et al., 2019), making it possible to deliver therapeutic agents for neurodegenerative diseases to the target site (Pardeshi & Belgamwar, 2013). High levels of mucus are present in the nasal mucosa, and nanoparticles need to move quickly through the mucus when nanoparticles are delivered by intranasal administration. PEG modification promotes movement through the mucus.

RVG29 is a 29-amino acid-derived peptide derived from the rabies virus glycoprotein. This protein can carry drugs and nicotinic acetylcholine receptors (NAchR) in nerve cells (Oswald et al., 2017) and can also cooperatively function with nerves. Cell adhesion molecules (NCAM) or p75 neurotrophin receptors (P75NTR) (Lafon, 2005) show highly efficient brain-targeted drug delivery, and their modification of drug carriers has been shown to be a safe strategy (Gan et al., 2019). Further reports on RVG29 have examined modified intravenous delivery vectors to improve brain targeting (Chen et al., 2011), but drugs need to pass through the blood-brain barrier to enter the brain from the circulatory system. However, RVG29 modification has been reported to increase drug accumulation in the brain tissue. Administration of the drug directly through nasal inhalation to avoid the blood-brain barrier may be a more efficient method. We believe that RVG29-modified nanoparticle-encapsulated nucleic acid drugs can enhance the ability of the olfactory and trigeminal nerves in the nasal mucosa to take up the drug, increase the drug levels near neural tissue, and improve entry through neuronal endocytosis or nerve cells in the brain. No reports have currently examined the administration of RVG29-modified nanoparticles by nasal inhalation to treat neurological diseases.

In this study, RVG29 was modified by the PEG-PLGA polymer, and miR-124 was encapsulated by the double emulsion method to form nanoparticles. Various physicochemical indicators showed that this strategy was suitable for nasal delivery. *In vivo* experiments showed that the RVG29-modified nanoparticles rapidly entered the brain tissue, and high levels were detected by a fluorescence tracer. Rats that underwent ischemic brain injury and were administered nucleic acid drugs via nasal absorption were examined through behavioral tests, and we verified the drug delivery through the nasal route. MiR-124 downregulated the RhoA protein levels and upregulated GAP43 expression in the rat brains to promote the pharmacodynamic effects on synaptic growth (Gu et al., 2018). In this study, modified PEG-PLGA nanoparticles were used to deliver miR-124 through intranasal delivery for the first time using high neurophilic protein 29 (RVG29), which can protect the nerve cells of the brain by altering the RhoA protein in a rat model of focal cerebral ischemia. These results suggest a new potential treatment for ischemic brain injury in the future.

## 2. Materials and methods

### 2.1. Preparation and physicochemical analyses of nanoparticles

#### 2.1.1. Materials

Mal-PEG-PLGA (maleimide-poly(ethylene glycol)-poly(lactic-co-glycolic acid)), MePEG-PLGA, and PLGA were purchased from Jinan Daigang Biomaterial Co., Ltd. Spermidine and DiR (1,1′-dioctadecyl-3,3,3′,3′-tetramethylindotricarbo-cyanine iodide) were purchased from Sigma-Aldrich (St. Louis, MO). RVG29-Cyspeptide and PLGA-maleimide (MAL) were purchased from Changchun Xinjinji Biological Technology Co., Ltd. The miR-124 mimic was purchased from Shanghai GenePharma Co., Ltd.

#### 2.1.2. Preparation and characterization of nanoparticles

PEG-PLGA nanoparticles loaded with miR-124 were prepared by a double emulsion method. MiR-124 was mixed with spermidine in a 10:1 N/P ratio in RNase-free water to form a complex. Eighteen milligrams of PEG-PLGA and 2 mg of maleimide-PEG-PLGA were separately dissolved in 2 mL of dichloromethane (DCM), and then, the spermidine/miR124 complex was added to the above DCM solution and sonicated for 60 s to form a primary emulsion. The primary emulsion was added to 20 mL of a 2.5% PVA (w/v) aqueous solution and stirred, and the organic solvent was removed by rotary evaporation. The nanoparticles were concentrated by centrifugation at 21,000 × *g* for 45 min and washed 3 times with deionized water. The maleimide-thiol interaction was used to couple the RGV29 peptide to the surface of the NPs. The excess RGV29 peptide was incubated for 8 h, and the unreacted peptide was removed by centrifugation (12,000 *g*/15 min). The same steps were used to prepare RVG29-modified PLGA nanoparticles and blank nanoparticles without miR-124. DiR-labeled nanoparticles were generated by adding DiR to the oil phase, and the preparation process was the same as described above. The particle size and zeta potential of the nanoparticles were measured by dynamic light scattering (DLS) using a Zetasizer 3000 (Malvern Instruments, UK). Nanoparticles were imaged using a transmission electron microscope (TEM; H-7650, Hitachi, Chiyoda, Japan). The encapsulation efficiency of miRNA loading was tested with a Quant-iT™ RiboGreen kit.

#### 2.1.3. Distribution in the brain after nasal delivery of the nanoparticles

Rats with MCAO were intranasally instilled with PEG-PLGA NPs and RVG29-PEG-PLGA NPs labeled with the dye 1,1′-dioctadecyl-3,3,3′3′-tetramethylindotricarbo-iodine cyanine (DiR). Two hours later, the heart was perfused with normal saline, and the brain and internal organs were collected. Biofluorescence imaging systems (IVIS Spectrum, Perkin Elmer) were used to capture and analyze tissue fluorescence images of the brain and related organs.

### 2.2. Studies of laboratory animals

#### 2.2.1. Source of animals

Adult SD rats (weight 250–300 g, age 12–15 weeks, sex ratio 1:1) were selected and provided by Liaoning Changsheng Biotechnology Co., Ltd. (animal production license number: SCXK (Liao) 2015-0001). The controlled temperature was 23 ± 2 °C, the relative humidity was 54 + 2%, and water was freely available. All operation procedures were in accordance with the requirement for ethical approval by the Ethics Committee of the School of Pharmaceuticals Sciences, Jilin University. All animal studies were strictly conducted under the guidelines of the ‘National Animal Management Regulations of China’ and were approved by the Animal Ethics Committee of the School of Pharmaceuticals Sciences, Jilin University.

#### 2.2.2. Establishment of a rat model of ischemic brain injury

We used the modified line embolus method for 2 h to generate the rat model of focal cerebral ischemia model (MCAO). Briefly, the rats were administered 10% chloral hydrate, and the common carotid artery, internal carotid artery, and external carotid artery were exposed. A surgical 4-0 nylon line was used to ligate the artery from the external carotid artery to the internal carotid artery (18.5–19.5), and the right middle cerebral artery was obstructed, inducing MCAO for 2 h. Then, the nylon line was removed. The neurological function scores were determined at 24 h, and the animals were divided into groups (see the preparation video in the Supplementary materials).

#### 2.2.3. Grouping and treatment of the experimental animals

Rats were divided into different groups and administered the appropriate treatments. Each group received a drug intervention before animal modeling. The specific groups were the sham, control (MCAO rats without treatment), and miR-124 nasal administration groups, including the PEG-PLGA/miRNA-124 nasal administration group, the RVG29-PEG-PLGA/miRNA-124 nasal administration group, and the RVG29-PLGA/miRNA-124 nasal administration group. Six animals were in each group. In each group, the administration was started 7 days before the modeling, and on the 7th, the 5th, the 3rd, and the 1st day of the modeling, each rat was nasally administered two drops of miRNA-124 at a concentration of 5 µg/mL aqueous solution. All rats were anesthetized and sacrificed after 3 days. All procedures complied with the relevant animal ethical regulations.

#### 2.2.4. Assessment of neurological deficits

After 24 h of ischemia/reperfusion in the rats, the Zea Longa scoring method was used to score the MCAO reperfusion model, and the scoring criteria were as follows:

- 0 score: no symptoms of nerve injury;
- 1 points: cannot fully extend the contralateral front claw;
- 2 points: rear-end turn to the opposite side;
- 3 points: slouching to the opposite side;
- 4 points: unable to walk spontaneously, loss of consciousness.

#### 2.2.5. Beam walking test

Fine motor coordination and balance were assessed by the beam walking assay. This test is particularly useful for detecting subtle deficits in motor skills and balance that may not be detected by other motor tests, such as the rotarod test. A beam-walking test was applied to assess motor coordination and balance control of the forelimbs and hindlimbs to evaluate neurological functional recovery in the rats after ischemia. Beam walking tests were used to assess fine motor coordination 24 h after MCAO. Briefly, each rat was trained to traverse a horizontal beam 2.5 cm wide, 80 cm long and 60 cm above the floor. Rats were motivated to cross the beam by placing their home cage at the other end. The behavioral response was scored as follows:

- unable to stay on the beam;
- 1, able only to stay on the beam;
- 2, tried to traverse the beam but fell:
- 3, traversed the beam with more than 50% hindlimb foot slips;
- 4, traversed the beam with fewer than 50% foot slips;
- 5, traversed the beam with only one slip;
- 6, traversed the beam with no slips.

#### 2.2.6. TTC staining to measure infarct volume

Cerebral infarction volume in rats was measured by TTC staining. The rats were anesthetized, and the cerebellum and olfactory bulb were removed. After the samples were frozen at −20 °C for 30 min, continuous coronal sections of the brain tissue were obtained at 2 mm intervals, stained with 1% TTC solution at a constant temperature of 37 °C for 30 min, and stained with regular rotation. The infarcted areas of the brain were white, and the noninfarcted areas were red. The section was photographed and analyzed by ImageJ software. Cerebral infarction area (%) = cerebral infarction area/whole brain area ×100%.

#### 2.2.7. Immunofluorescence test, Western blot detection of RhoA and GAP43

Some of the rats in each group were subjected to heart perfusion with paraformaldehyde. After the brain tissue was fixed, the brain was stored in a 4% paraformaldehyde solution for subsequent immunofluorescence detection. After the brains of the remaining groups of rats were collected, immunofluorescence was used to assess RhoA and GAP43. A portion of the brain tissue was directly frozen in liquid nitrogen for PCR and Western blot analyses of RhoA and GAP43, and RhoA and GAP43 were analyzed.

### 2.3. Data analysis

One-way analysis of variance and Newman–Keuls tests were used for the statistical analyses. Statistical analysis was performed using GraphPad Prism 6.0 software. Quantitative analysis of the WB bands was performed using ImageJ analysis software.

## 3. Results

### 3.1. Nanoparticle characterization

**Figure 1.**
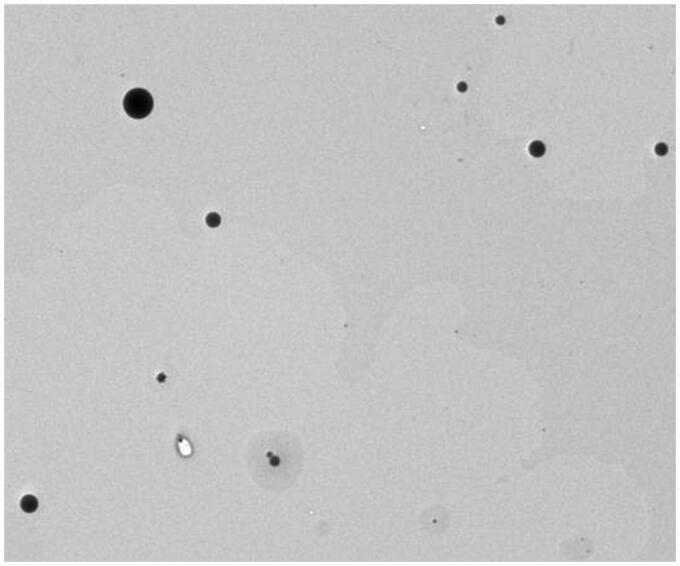
Transmission electron microscopy (TEM) of nanoparticles.

Transmission electron microscopy (TEM) analysis showed that the RVG29-PEG-PLGA/miRNA-124 nanoparticles appeared regular and spherical (Figure 1). The average diameter of the prepared RVG29-PEG-PLGA/miRNA-124 nanoparticles was 204 nm, and the polydispersity index (PDI) was less than 0.4, indicating that the prepared nanoparticles have a narrow size distribution. Compared with that of PEG-PLGA/miRNA-124, the particle size of RVG29-PEG-PLGA/miRNA-124 was slightly larger, and the zeta potential was slightly increased. The nanoparticle miRNA encapsulation efficiency was 28.2 ± 8.3% (Figure 1).

### 3.2. Fluorescence comparison of the nanoparticles in the tissues

**Figure 2.**
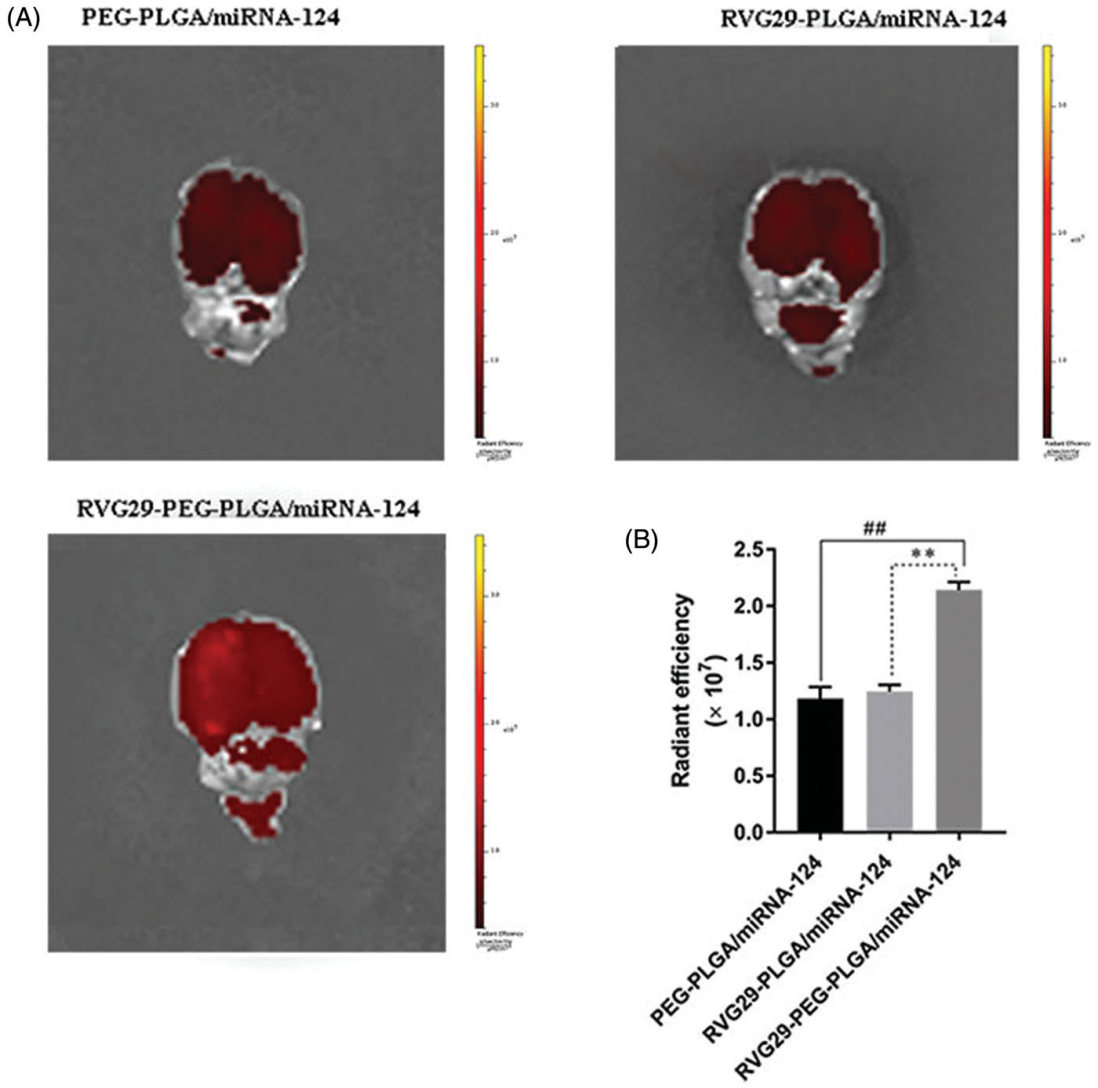
Brain distribution of PEG-PLGA/miRNA-124, RVG29-PLGA/miRNA-124 and RVG29-PEG-PLGA/miRNA-124 following intranasal administration in the stroke model. *Ex vivo* fluorescence brain images (A) and summarized data (B). ^##^*p* < .01 compared with the RVG29-PEG-PLGA/miRNA-124 group; **p < .01 compared with the RVG29- PLGA/miRNA-124 group.

In order to assess the aggregation of nanomaterial coated miRNA-124 in the brain of ischemic stroke, after successful modeling of ischemic stroke, three kinds of nanoparticles (DiR labeled peg-plga/miRNA-124, rvg29-plga/miRNA-124, and rvg29-peg-plga/miRNA-124) were administered to the nasal mucosa, and the fluorescence intensity in the brain was detected 4 h after administration. The results showed that rvg29-peg-plga/miRNA-124 had the highest accumulation of fluorescence intensity in the brain compared with the other two groups. The accumulation of peg-plga/miRNA-124 and rvg29-plga/miRNA-124 in the brain was small and the differences were not significant. It indicated that the selection of nanoparticle ascomaterials and the existence of modification groups can help the nanoparticle to reach the target position better. The experimental results showed that peg-plga modified with RVG29 could be used as a nanocarrier to enhance the aggregation of the drug in the brain (Figure 2).

### 3.3. Neural function score after modeling

After MCAO modeling was completed, 2 rats in the ischemic brain injury group died and were replaced. The neurological deficit scores in the ischemic brain injury group and the miRNA-administered groups were significantly higher than those in the sham operation group (*p* < .05), showing that the miRNA-administered groups displayed improvements in brain function compared to the model group. The neurological deficit scores of RVG29-PEG-PLGA/miRNA-124 nasal administration group were significantly lower than those of the ischemic brain model group (*p* < .05) but higher than those of the sham operation group. The nanoparticle group-administered miRNA by nasal inhalation had the lowest neurological defect score, indicating the excellent therapeutic effect of this treatment (Table 1).

**Table 1.** The neurological score of MCAO rats treated with intranasal administration of nanoparticles.

Groups	score
Sham groups	0.00 ± 0.00
Control groups	3.50 ± 0.55
MiR-124 nasal administration groups	2.83 ± 0.41
PEG-PLGA/miRNA-124 nasal administration group	2.67 ± 0.82
RVG29-PLGA/miRNA-124 nasal administration group	1.83 ± 0.75
RVG29-PEG-PLGA/miRNA-124 nasal administration group	1.00 ± 0.63

### 3.4. Beam balance test (BBT)

The beam balance walking test was used to detect the recovery of motor nerve function in the rats. The results showed that compared with that of the sham operation group (5.83 ± 0.41), the percentage error on the beam balance in the ischemic brain injury group was significantly higher (1.67 ± 0.82, *p* < .01). Compared with that in the control group, the percentage error on the beam balance was significantly reduced the PEG-PLGA/miRNA-124 nasal administration group (3.33 ± 0.52, *p* < .01) Compared with that in the PEG-PLGA/miRNA-124 nasal administration group, the percentage error on the beam balance was significantly reduced RVG29-PEG-PLGA/miRNA-124 nasal administration group (5.00 ± 0.63, *p* < .05). These results showed that the nasal inhalation method is very effective and fast for treating brain diseases and can significantly reduce dyskinesia after cerebral infarction. The carrier form of the nanoparticles and miRNA had the best pharmacodynamic effect (Table 2).

**Table 2.** The beam balance test score of MCAO rats treated with intranasal administration of nanoparticles.

Groups	The first-day score	The third-day score
Sham groups	5.67 ± 0.52	5.83 ± 0.41
Control groups	1.50 ± 0.84	1.67 ± 0.82
MiR-124 nasal administration groups	2.33 ± 0.52	2.50 ± 0.55
PEG-PLGA/miRNA-124 nasal administration group	3.17 ± 0.75	3.33 ± 0.52
RVG29-PLGA/miRNA-124 nasal administration group	4.00 ± 0.63	4.17 ± 0.41
RVG29-PEG-PLGA/miRNA-124 nasal administration group	4.83 ± 0.75	5.00 ± 0.63

### 3.5. TTC test results

**Figure 3.**
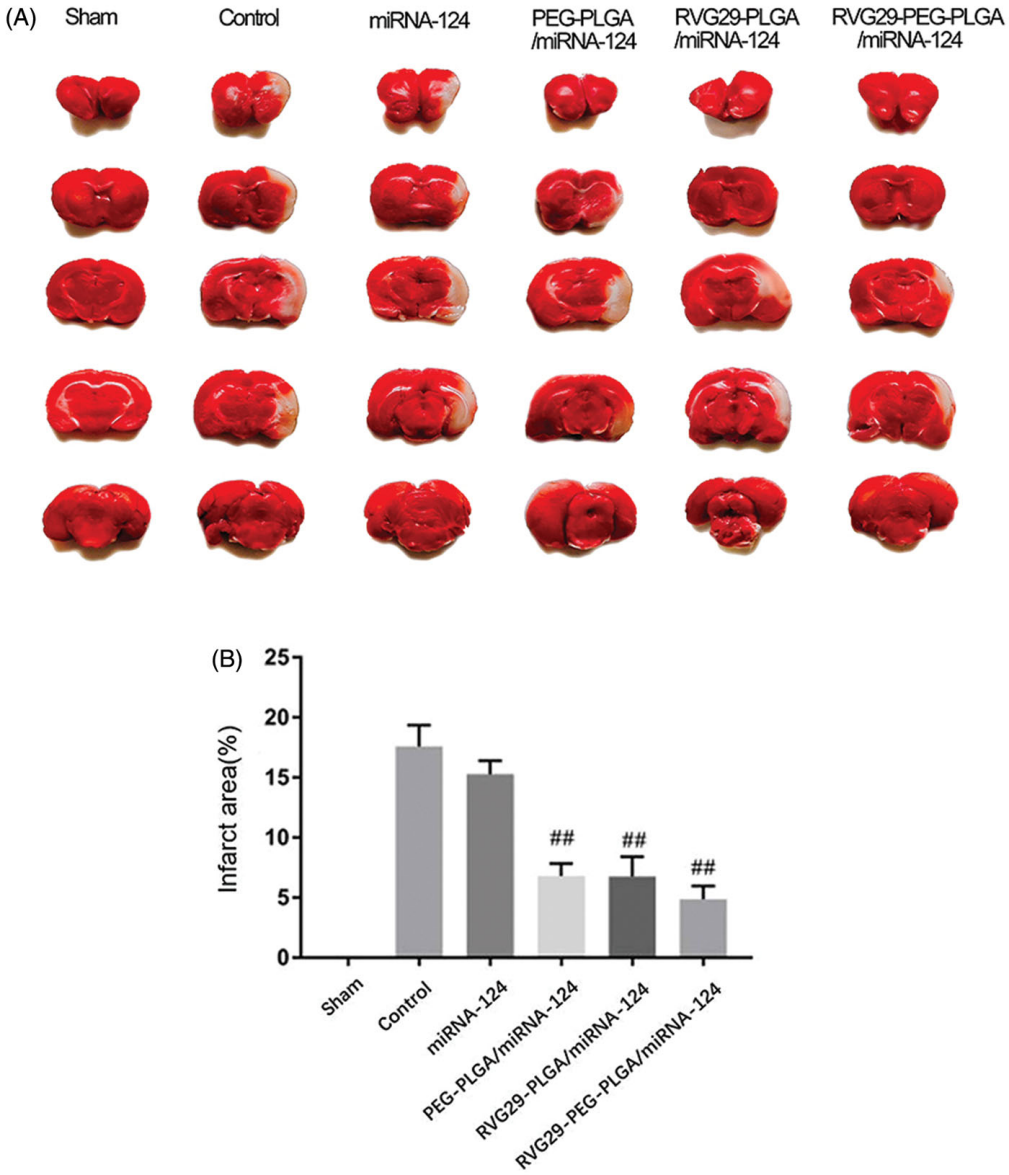
RVG29-PEG-PLGA/miRNA-124 on the cerebral infarction volum. TTC staining (A) and the quantification (B) of the ischemic brain injury of MCAO rats. ^##^*p* < .01 compared with the Control group.

TTC tests were used to verify the effect of nasal administration of RVG29-NPs-miR124 on cerebral ischemia-reperfusion. TCC staining was used to assess the size of the cerebral infarction area; the lesion area of the cerebral infarction was not stained white. The MCAO treatment lasted 2 h, and the brain infarct size and the neurological score were measured at 24 h after reperfusion. As shown in Figure, the infarct area of the model group and the RVG29-NP group was basically the same, and the neurobehavioral score did not show significant improvements, indicating that the nanoparticles themselves had no therapeutic effect; however, the administration of RVG29-NP/miR-124 improved the neurobehavioral score and significantly reduced the infarct size. Naked miR-124 had an effect on the infarct area, but this effect was not optimal. These results suggest that nanoparticles can protect miR-124 from nasal cilia or related enzymes to a certain extent (Figure 3).

### 3.6. Rhoa and GAP43 expression levels in rats were detected by immunofluorescence

**Figure 4.**
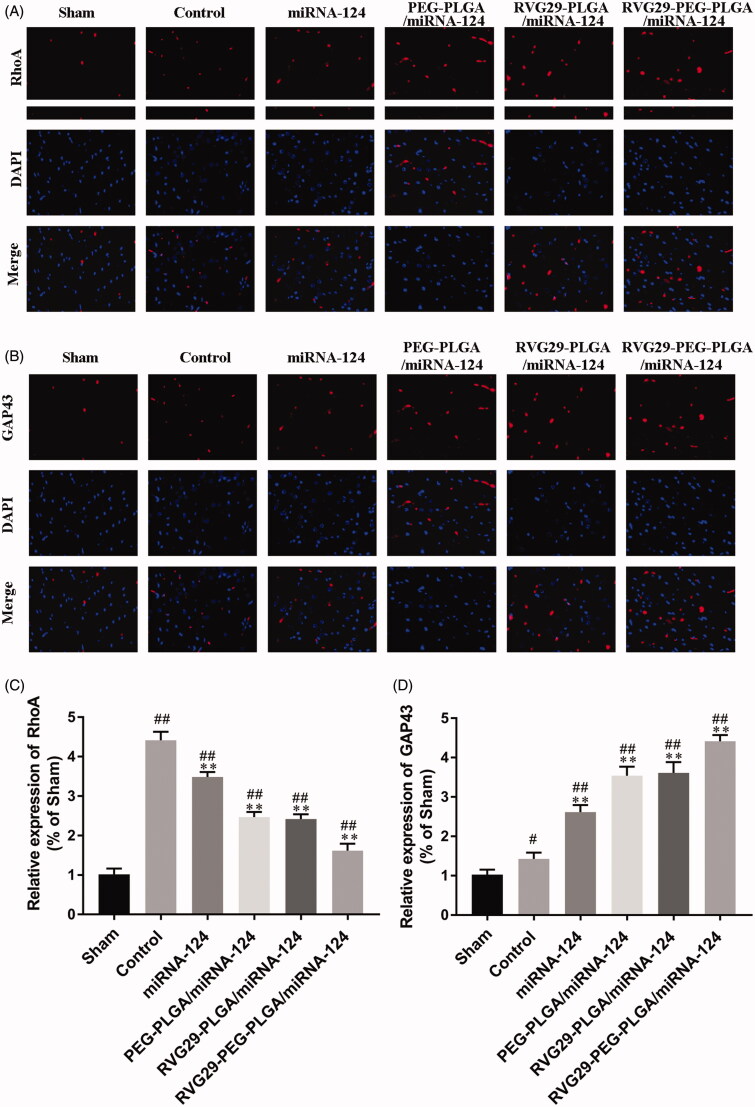
Representative immunofluorescence images showing RhoA and GAP43. Representative images depict the immunolabeling of RhoA (A) and GAP43 (B) in the cerebral cortex and quantitative analysis of RhoA (C) and GAP43 (D) reveals their expression in the mouse cerebral cortex. ^#^*p* < .05, ^##^*p* < .01 compared with the Sham group; **p < .01 compared with the Control group.

RhoA is the main scaffold protein in excitatory synapses and postsynaptic densities and has been characterized as one of the most abundant scaffold proteins in excitatory neurons. GAP43 is a marker of neurons. Quantitative analysis showed that the fluorescence intensities of RhoA and GAP43 in the RVG29-NPs-miR124 group were significantly higher than those of the MCAO model group. primarily due to the high-affinity interaction of the RVG29 conjugated to the nanoparticle surface with the neuron surface receptors, and the nanoparticle protected miR-124 from being degraded and could reach the target site through nasal inhalation (Figure 4).

### 3.7. Rhoa and GAP43 expression levels in rats were detected by Western blots

**Figure 5.**
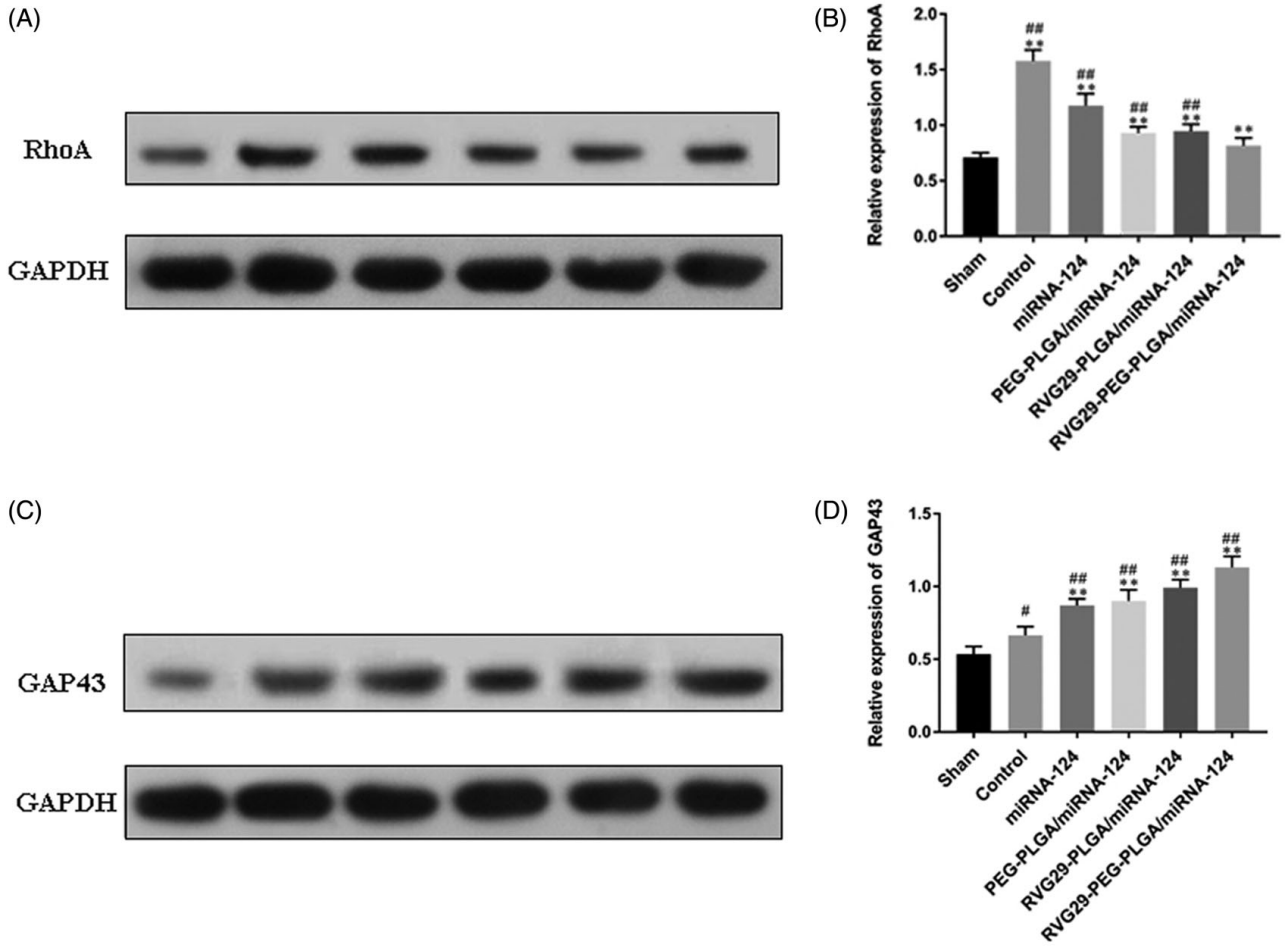
Effect of miR-124 administration on protein expression of RhoA and GAP43 after MCAO. (A) RhoA and (C) GAP43 protein were detected by Western blot. GAPDH was used as the loading control. Quantitative analysis of RhoA (B) and GAP43 (D) activity. ^#^*p* < .05, ^##^*p* < .01 compared with the Sham group; **p < .01 compared with the Control group.

Changes in protein expression were detected by Western blots. The results showed that the expression levels of RhoA and GAP43 increased in the MCAO group. The RVG29-NPs-miR124 group exhibited significantly decreased expression of RhoA in the Area of cerebral infarction. Furthermore, GAP43 expression was increased compared with that in the other groups. In the PEG-PLGA group and the RVG29-PLGA group, the upregulation of GAP43 was weaker than that in the RVG29-PEG-PLGA group. These results showed that the modification of RVG29 and PEG is important to enhance the nasal inhalation of the PLGA nanoparticles (Figure 5).

## 4. Discussion

Ischemic cerebral apoplexy is the second most fatal disease worldwide after cardiovascular disease (Heuser, 2017). This condition is mostly caused by cerebral vascular embolism due to the rupture of arterial plaque and may also be related to atrial fibrillation, sudden hypercoagulability, vasospasm, hemodynamic changes, and other factors. Currently, little progress has been made in treating this disease based on the etiology (Fonseca & Ferro, 2015). Clinically, the main purpose of treatment is to quickly restore nerve function after the onset of disease.

When an ischemic brain injury occurs, the cytoskeleton needs to be regulated after the nervous system is damaged to maintain the dynamic stability between different components of neurons to achieve the integrity of the structure and function of nerve cells (Fujita & Yamashita, 2014). As the main mechanical structure of eukaryotic cells, the cytoskeleton plays an important role in maintaining the morphology and function of nerve cells, especially in maintaining the morphology of damaged nerve tissues, tolerating external forces and maintaining the order of the internal structure of nerve cells (Eira et al., 2016). As the downstream target of GTPase Rho, Rho-kinase is a serine and threonine kinase, and RhoA plays a role in regulating the nerve cytoskeleton (Escamilla et al., 2017).

After cerebral ischemia, RhoA expression increases in the brain, strengthening the polymerization of actin in the growth cone and driving axial stretch to cause synaptic contraction of dendritic and spinous processes in nerve cells (Mulherkar et al., 2017). These changes result in a functional synaptic reduction, growth cone collapse, and accelerated nerve necrosis (Park et al., 2017). Therefore, inhibition of the RhoA signaling pathway can quickly prevent further destruction of nerve cell polarity and further decreases in the ability of nerves to transmit electrical impulses and neuronal information (Kim et al., 2017). This study showed that the miRNA group treated with nasal aspiration could more effectively reduce the expression of RhoA and maintain the damaged nerve cytoskeleton than the other groups.

With the development of research, interventions for various diseases at the genetic level are increasingly recognized. MiRNAs were originally discovered in *Caenorhabditis elegans* (Lee et al., 1993); this class of small RNA molecules of ∼20 nucleotides mainly functions by inhibiting the translation and degradation of the target mRNAs and can thus influence related protein molecule expression due to the specificity of gene silencing. Therefore, these molecules can regulate various diseases. The advantage of miRNA lies in the regulation of gene networks. Currently, ∼50% of miRNAs exist in the brain, which is in a highly enriched and relatively conservative state (Piwecka et al., 2017), playing an important role in the development of the nervous system, maintenance of neurophenotypes and neuroprotection of ischemic brain injury (Saugstad, 2010). It has been reported that miRNA-124, miRNA204, miRNA501-3p (Hu et al., 2015), miRNA155, miRNA134, and miRNA137 all have neuroprotective effects in ischemic brain injury (Danka Mohammed et al., 2017), and miRNA-124 was shown to affect the RhoA signaling pathways that play a role in nerve protection. MiRNA-124 has been reported in Alzheimer’s disease (An et al., 2017), Parkinson’s disease (Yao et al., 2018), epilepsy (Brennan et al., 2016), depression (Higuchi et al., 2016), etc.

Our results showed that nasal administration of miRNA-124 for ischemic brain injury had significant effects. As an abundant neuron-specific miRNA in the brain, under different conditions, miR-124 has been identified as a promoter of neuronal generation in vitro and *in vivo*, with potential regulatory effects on postmitotic neuronal activity, such as synaptic plasticity and memory formation (Maiorano & Mallamaci, 2010). After the occurrence of acute ischemic stroke, the content of miR-124 in human serum exosomes will increase (Ji et al., 2016). Based on the synchronous increase in miR-124 in brain tissue and plasma, miR-124 can be used as a biomarker for stroke (Wang et al., 2018). This molecule promoted hippocampal neurogenesis after brain injury and improved functional recovery after cerebral ischemia and reperfusion (Yang, Ye, et al., 2019). MiR-124 plays a role in the synaptic plasticity of nerve cells (Hou et al., 2015), promoting the proliferation and differentiation of neural stem cells (Jiao et al., 2018). Upregulation of miR-124 can reduce the area of cerebral infarction, promote the recovery of nerve function after ischemic stroke (Huang et al., 2019), and improve spatial learning ability and neuronal activity in the hippocampus (Yang, Guo, et al., 2019). MiR-124 plays a key role in neuronal plasticity in rats with cerebral infarction (González-Giraldo et al., 2015), promoting neuronal proliferation (Wang, Wang, et al., 2019), inhibiting apoptosis (Che et al., 2019), and promoting synaptic elongation and elongation of neurons in the young animals (Su et al., 2020). MiR-124 may be involved in the regulation of neurite outgrowth by regulating the cytoskeleton (Yu et al., 2008).

RhoA has a negative regulatory effect on neuronal synapse development (Walchli et al., 2013) Regulating the mRNA targets of RhoA in the brain can regulate synaptic remodeling of brain neurons and improve learning and memory (Berger et al., 2017). MiR-124 directly targets the RhoA mRNA and inhibits its expression in neurogenesis (Wang et al., 2016).

GAP43 is a marker of neurite elongation and synapse formation (Rosskothen-Kuhl & Illing, 2014) is a protein involved in neurite outgrowth and axon regeneration (Gorup et al., 2015) and plays an important role in regulating nerve buds and actin skeletons under the regulation of RhoA (Wang, Li, et al., 2019). The increased expression of GAP43 is a sign of neural regeneration (Sanna et al., 2014). After cerebral apoplexy, the high expression of GAP43 in the infarcted area of the miR-124 group receiving nasal inhalation indicates that the axons around the infarcted area may recover. Although the administration of miR-124 has been shown to protect the nerves, first-pass effects such as gastrointestinal metabolism, liver breakdown, and the blood-brain barrier reduce the drug levels reaching the site of damaged neurons. In addition, nucleic acid drugs have the disadvantage of being easily degraded by enzymes. Therefore, we combined miR-124 with nanodrug carrier technology to avoid tissue degradation of the drugs. Furthermore, noninvasive intranasal delivery can improve the brain targeting of drugs and reduce systemic toxicity (Pires & Santos, 2018). At present, the commonly used nanodrug carriers are lentiviral vectors and synthetic polymer-carriers. Considering that viral vectors have certain side effects and that target modification is relatively difficult, we choose artificially synthesized PEG-PLGA as a carrier. The carrier has a simple synthetic process, controllable size, low immunogenicity, easy modification and preservation, good biocompatibility, and high reproducibility and can protect large molecules, which can affect the release rate (Ding & Zhu, 2018; Park et al., 2018). In recent years, various types of nanocarriers, such as polymers, emulsions, lipid carriers, carbon nanotubes, and metal-based carriers, have been used as slow-release carriers for drugs (Karthivashan et al., 2018). This carrier is widely used to encapsulate conventional drugs to improve fat solubility and controlled release effects (Yu et al., 2017), encapsulate gene drugs, reduce cytotoxicity, and increase delivery efficiency and drug modification properties (Ramezani et al., 2017).

The nasal-to-brain drug delivery pathway occurs via nasal breathing and the olfactory area through the intracellular exocytosis of cells or neurons or the intercellular pathway along the cells near the olfactory nerve, and the transcellular pathway across basal epithelial cells mediates drug delivery to the brain (Hong et al., 2019) One advantage is the ability to avoid the blood-brain barrier (Crowe et al., 2018) For example, it has been reported that the delivery of insulin selection-related vectors through the nasal route to the brain can increase the level of insulin in the brain parenchyma to improve Alzheimer’s disease (Kamei et al., 2017) The olfactory region of the nasal mucosa has a high degree of nerve enrichment. To enhance the ability of drugs to reach the olfactory region, we modified the drug carrier with RVG29 to enhance its affinity to nerves. RVG29 has a neurogenic affinity, and an intravenous drug modified by RVG29 binds tightly to endothelial cells in the brain (Hua et al., 2018)he brain-targeting properties of Rvg29 can mediate passage across the blood-brain barrier and are widely used to modify neurotherapeutics for treatment of Parkinson’s disease, neuroinflammation, and gliomas (Gan et al., 2019; Chen et al., 2011; You et al., 2018) This vehicle can interact with nerve cell-associated neural cell adhesion molecules (NCAM), p75 neurotrophin receptor (P75NTR) (Gu et al., 2018), and nicotinic acetylcholine receptor nAChRs (Lafon, 2005; Cook et al., 2015) and it shows neural targeting (Huey et al., 2017) Nanodrugs can be modified to improve the efficiency of drug delivery through the nose to the brain (Feng et al., 2018). In our experiments, we found that the administration of drugs through the nasal mucosa did reach the brain. The nose-brain osmotic mechanism of nanoparticles is that the nanoparticles pass through the nasal mucosa and nasal epithelial cells after the nanoparticles are given intranasally, reach the vicinity of the olfactory nerve and trigeminal nerve, and enter the brain with the nerve. The targeted part of the olfactory nerve pathway is located in the olfactory bulb, trigeminal nerve pathway targets brain tail, we observed a large amount of fluorescence display in the dorsal side of the brain and a small amount of fluorescence display in the cerebellar region of the tail of the rats. Therefore, we determined that the nanoparticles mainly reached the olfactory bulb through the olfactory nerve pathway, and a small part of them reached the cerebellum through the trigeminal nerve pathway and then integrated into the cerebrospinal fluid to form the fluorescence distribution in the dorsal brain and cerebellar regions in this experiment (Figure 2).

## 5. Conclusion

In this study, we verified our hypothesis that miR-124 encapsulated in RVG29-PEG-PLGA nanoparticles and administered through the nose, which allowed the drug to enter the brain and affect the expression of RhoA, could produce neuroprotective effects. The modification of RVG29 and PEG improved the effect of the PLGA nanoparticles as the delivery carrier through nasal inhalation. Therefore, we believe that supplementation with RVG29 and nasal delivery of miR-124 can indeed contribute to the recovery of neurological function after cerebral ischemia.
